# New York State Veterinary Medical Society

**Published:** 1896-05

**Authors:** 


### NEW YORK STATE VETERINARY MEDICAL SOCIETY.

*Officers and Committees Elected at New York City, Sept. 5 and 6, 1895.*
President, Nelson P. Hinkley, 395 Ellicott St., Buffalo; Vice-President, Thomas Giffen, 217 W. Fifty-eighth St., New York City; Secretary-Treasurer, Claude D. Morris, Pawling.

Board of Censors: W. L. Baker, Cortland; John Wende, 1595 Main St., Buffalo; R. S. Huidekoper, 154 E. Fifty-seventh St., New York City; Arthur O'Shea, 117 W. Forty-sixth St., New York City; Prof. James Law, Ithaca (Cornell University).

Committee of Arrangements: Claude D. Morris, Pawling; L. A. Robinson, Buffalo; George E. Gangloff, Buffalo; L. E. Willyoung, Buffalo; Samuel Somerville, Jr., Buffalo; J. P. Thompson, Niagara Falls.

Committee on Legislation: Nelson P. Hinkley, Buffalo; Claude D. Morris, Pawling; William Henry Kelly, Albany; James Law, Ithaca; Arthur O'Shea, New York City.

Committee on By-Laws: Claude D. Morris, Pawling; Wilson Huff, Rome; John A. Bell, Watertown.

Executive Committee: Nelson P. Hinkley, Buffalo; Thomas Giffen, New York City; Claude D. Morris, Pawling; L. E. Willyoung, Buffalo; William Henry Kelly, Albany; Wilson Huff, Rome.

County Secretaries: John A. Bell, Watertown, Jefferson Co.; W. L. Baker, Cortland, Cortland Co.; E. H. Hayden, Syracuse, Onondaga Co.; James Carnrite, Amsterdam, Montgomery Co.; Charles Cowie, Ogdensburg, St. Lawrence Co.; W. J. Wadsworth, Cobleskill, Schoharie Co.; J. M. Chase, Seneca Falls, Seneca Co.; E. L. James, Cooperstown, Otsego Co.; W. H. Carpenter, Johnstown, Fulton Co.; R. D. Austin, Schenectady, Schenectady Co.; J. A. Genung, Ithaca, Tompkins Co.; O. B. French, Honoeye Falls, Monroe Co.; George D. Dowland, Auburn, Cayuga Co.; Wilson Huff, Rome, Oneida Co.; W. E. Stocking, Eagle Harbor, Orleans Co.; James McKee, Stapleton, Richmond Co.; E. H. Nodyne, Sodus, Wayne Co.; M. M. Poucher, Oswego, Oswego Co.; W. B. Ingalls, Mohawk, Herkimer Co.; D. S. Saylor, Glen Falls, Warren Co.; Arthur O'Shea, New York City, New York Co.; C. D. Morris, Pawling, Dutchess Co.; J. P. Thompson, Niagara Falls, Niagara Co.; William H. Kelly, Albany, Albany Co.; J. K. Sutterby, Le Roy, Genesee Co.; John A. Huhne, Kingston, Ulster Co.; W. D. Ambler, Chatham, Columbia Co.; George H. Berns, Brooklyn, Kings Co.; H. McWhinnie, Troy, Rensselaer Co.; L. E. Willyoung, Buffalo, Erie Co.; E. Lalowell, Yonkers, Westchester Co.; J. L. Ronan, Corning, Steuben Co.; R. C. Jones, Port Jefferson, Suffolk Co.

